# Nomograms confirm serum IL-6 and CRP as predictors of immune checkpoint inhibitor efficacy in unresectable hepatocellular carcinoma

**DOI:** 10.3389/fimmu.2024.1329634

**Published:** 2024-01-18

**Authors:** Jiajia Du, Zhiyong Huang, Erlei Zhang

**Affiliations:** Hepatic Surgery Center, Tongji Hospital, Tongji Medical College, Huazhong University of Science and Technology, Wuhan, China

**Keywords:** IL-6, CRP, HCC, ICIs, prognosis

## Abstract

**Background:**

Immunotherapy based on immune checkpoint inhibitors (ICIs) has become the first-line treatment for unresectable hepatocellular carcinoma (uHCC). However, only a small portion of patients are responsive to ICIs. It is important to identify the patients who are likely to benefit from ICIs in clinical practice. We aimed to examine the significance of serum IL-6 and CRP levels in predicting the effectiveness of ICIs for uHCC.

**Methods:**

We retrospectively recruited 222 uHCC patients who received ICIs treatment (training cohort: 124 patients, validation cohort: 98 patients). In the training cohort, patients are categorized into the response group (R) and no-response group (NR). The levels of serum IL-6 and CRP were compared between the two groups. Internal validation was performed in the validation cohort. Survival analysis was carried out using the Kaplan-Meier method and Cox proportional hazard regression model. The nomograms were developed and assessed using the consistency index (C-index) and calibration curve.

**Results:**

Serum levels of IL-6 and CRP were significantly lower in the R group than in the NR group (9.94 vs. 36.85 pg/ml, p< 0.001; 9.90 vs. 24.50 mg/L, p< 0.001, respectively). An ROC curve was employed to identify the optimal cut-off values for IL-6 and CRP in both groups, resulting in values of 19.82 pg/ml and 15.50 mg/L, respectively. Multivariate Cox regression analysis revealed that MVI (HR 1.751, 95%CI 1.059-2.894, p=0.029; HR 1.530, 95%CI 0.955-2.451, p=0.077), elevated IL-6 (HR 1.624, 95%CI 1.016-2.596, p=0.043; HR 2.146, 95%CI 1.361-3.383, p =0.001) and high CRP (HR 1.709, 95%CI 1.041-2.807, p=0.034; HR 1.846, 95%CI 1.128-3.022, p = 0.015) were independent risk factors for PFS and OS, even after various confounders adjustments. Nomograms are well-structured and validated prognostic maps constructed from three variables, as MVI, IL6 and CRP.

**Conclusion:**

Low levels of IL-6 and CRP have a positive correlation with efficacy for uHCC patients receiving ICIs.

## 1 Introduction

Hepatocellular carcinoma (HCC) is the primary cause of cancer mortality globally (1). The majority of patients are diagnosed at later stages and ought to receive systemic therapy in agreement with clinical guidelines. Even with early detection, the recurrence rate after surgery is approximately 70% within 5 years (2), necessitating systemic treatment. Over recent years, the application of multi-target tyrosine kinase inhibitors (TKIs) like Sorafenib and Lenvatinib has mitigated the survival of HCC patients to some extent; nevertheless, it falls notably short of clinical expectations. A combination of Atezolizumab and Bevacizumab, hinging on the IMbrave150 trial, was endorsed in 2020, demonstrating noteworthy enhancements in overall survival (OS), progression-free survival (PFS), and quality of life compared to sorafenib (3). Targeted therapy combined with immunotherapy is currently a viable first-line systemic treatment option. While patients who respond positively to this combined therapy tend to have good clinical outcomes, there are still individuals who exhibit significant resistance (4). As a result, there is an acute need to develop ICIs treatment effect predictors that will aid in identifying patient populations who would benefit.

Tumor tissue biomarkers, such as tumor-infiltrating lymphocyte counts and PD-1/PD-L1 expression, have been documented in various cancer types as indicators of the effectiveness of immune checkpoint inhibitors (ICIs). However, there is currently a lack of reliable biomarkers to predict the efficacy of immunotherapy in HCC. Moreover, patients with advanced HCC who are eligible for immunotherapy often undergo imaging tests instead of tumor biopsies, unlike patients with other solid cancers. Therefore, the development of non-invasive blood biomarkers is crucial. Existing predictors are often too invasive for widespread use, so we need to find simple, effective, and non-invasive methods. Many studies have proved serum inflammatory factors is promising. Neutrophils and platelets have been implicated in cancer immune-escape and progression trough cytokines productions (IL-18, VEGF, and PDGF) (5). M2 macrophages can promote the proliferation, migration, angiogenesis and immunosuppression of hepatocellular carcinoma cells (6).

Interleukin-6 (IL-6) is a cytokine produced by a variety of cell types, including immune cells, non-fibroblasts, endothelial cells, and tumor cells. IL-6 is upregulated in a variety of malignant tumors and plays a vital role in tumorigenesis and development by influencing tumor cell survival, proliferation, angiogenesis, invasiveness, and metastasis. In the liver, IL-6 levels gradually increase from a healthy state to hepatitis, cirrhosis and HCC (7). In addition, recent studies have identified resistance mechanisms to immunotherapy via the IL-6 signaling pathway (8). Overactivation of the IL-6 pathway may weaken the Th1 response and hinder T cell recruitment in the lymph nodes and the tumor microenvironment (TME), consequently suppressing anti-tumor T cell immunity (9). C-reactive protein (CRP) is an acute phase protein whose levels are elevated in an inflammatory state. It is primarily synthesized by the liver, with smaller amounts also produced by smooth muscle cells, macrophages, endothelial cells, lymphocytes, and fat cells in response to IL-6, TNF, and IL-1β. Recent research has revealed that pre-treatment CRP levels are significantly linked with PFS and OS in lung cancer patients treated with PD-1 inhibitors (10). This is consistent with published findings on hepatocellular carcinoma that indicates poor clinical outcomes in patients with high levels of serum acute phase proteins including CRP who are treated with PD-1 inhibitors (11). Increasing envidence has indicated that CRAFITY score, based on CRP and AFP levels, is a predictor of prognosis in HCC patients treated with immunotherapy (12, 13).

Herein, we conducted an analysis of peripheral blood samples obtained from patients diagnosed with hepatocellular carcinoma. Our objective was to investigate the potential correlation between serum levels of IL-6 and CRP with both survival rates and treatment response. Furthermore, we aimed to assess the prognostic value and predictive significance of elevated IL-6 and CRP levels in patients undergoing immunotherapy. Additionally, we examined the relationship between CRP and IL-6.

## 2 Patients and methods

### 2.1 Study design and patients

This retrospective study enrolled a total of 222 patients diagnosed with unresectable advanced HCC who underwent immunotherapy at Tongji Hospital in Wuhan between November 2020 and May 2023. Baseline blood samples and clinical data were collected from these patients. The study adhered to the guidelines outlined in the Helsinki Declaration and received approval from the hospital’s Ethics Committee (TJ-IRB20230866). Given the retrospective design of the study, the committee waived the need for informed consent from all patients.

**Figure 1:**
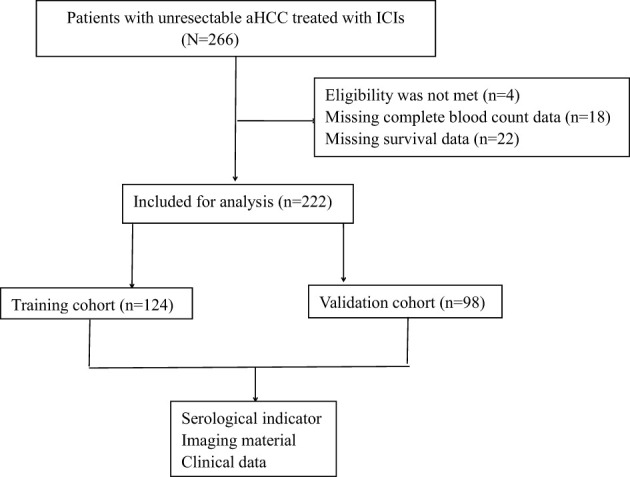
Data collected on the diagnosis of unresectable advanced hepatocellular carcinoma.

The study was conducted in two phases. In the first phase, we aim to identify meaningful biomarkers by analyzing clinically relevant biomarkers, including various cytokines, from the training cohort of 124 patients. In the second phase, the aim was to validate the clinical significance of these biomarkers in a separate validation group of 98 patients with uHCC who were undergoing immunotherapy (**Figure 1**).

The inclusion criteria were described below: 1) age 18–75 years; 2) expected survival >6 months, and ECOG score 0–1; 3) Child–Pugh 5–7 points; 4) BCLC stage: B and C; 5) neutrophil ≥1.5 × 10^9^/L, lymphocyte ≥0.5 × 10^9^/L, hemoglobin ≥90 g/L, and platelet ≥75 × 10^9^/L; 6) aspartate aminotransferase and alanine aminotransferase ≤3× the upper limit of normal value (ULN) and total bilirubin ≤3× the ULN; 7) international normalized ratio ≤1.5× the ULN; 8) creatinine ≤1.5* the ULN; 9) prior radiation therapy (RT), transcatheter arterial chemoembolization (TACE), hepatic arterial infusion chemotherapy (HAIC), and radiofrequency ablation (RFA) are permissible; and 10) follow-up targeted, surgical, RT, TACE, HAIC, and RFA are permissible.

The exclusion criteria were as follows: 1) previous chemotherapy, targeted therapy, immunization, surgery, and other systematic treatments; 2) individuals received antibiotics, hormones, immunosuppressants, aspirin, and atorvastatin 1 month before or during targeted therapy and immunotherapy; 3) acute inflammation, diabetes, hyperlipidemia, kidney disease, rheumatic disease, thyroid disease, autoimmune disease, or immune deficiency; 4) distant metastasis (except lymph node metastasis); and 5) malignancy with other sources.

The diagnosis of HCC relies on the imaging techniques and blood tumor markers. Intravenous administration of ICIs occurs every three weeks for a duration of one year or until disease progression and intolerable adverse reactions arise. Blood samples are collected prior to each cycle of ICIs injections. The evaluation of tumor size changes is conducted every six weeks using enhanced CT or MRI scans, employing both mRECIST and RECIST1.1 criteria. In the training cohort, patients are categorized into two groups based on treatment response. The response group (R) includes patients who have achieved complete response (CR), partial response (PR), and stable disease (SD) with a PFS exceeding six months. On the other hand, the non-response group (NR) consists of patients who have experienced disease progression (PD) or stable disease (SD) with a PFS of six months or less. Adverse reactions are assessed in accordance with the Common Terminology Criteria for Adverse Events (CTCAE) version 5.0. In the event of a treatment-related grade 3 to 4 adverse event, ICIs therapy will be temporarily halted, and the decision to resume immunotherapy will be made by at least two attending physicians.

According to the dual criteria of mRECIST and RECIST1.1, CR is characterized by the complete disappearance of all target lesions, the absence of any new lesions, and the presence of normal tumor markers for a minimum duration of 4 weeks. PR is defined as a reduction of at least 30% in the combined maximum diameters of all target lesions, sustained for a minimum of 4 weeks. SD is described as a reduction in the combined maximum diameters of the target lesions that does not meet the criteria for PR, or an increase that does not meet the criteria for PD. PD is defined as an increase of at least 20% in the combined maximum diameters of all target lesions, or the appearance of new lesions.

In this study, OS was determined by calculating the time from the initiation of ICIs treatment to either the date of death from any cause or the last follow-up. PFS was defined as the duration from the start of ICIs treatment to the occurrence of disease recurrence or metastasis. The objective response rate (ORR) was calculated as the percentage of patients whose tumors exhibited a certain degree of shrinkage and maintained that response for a specific period, including those with CR and PR. The disease control rate (DCR) was determined by considering the proportion of patients with CR, PR, and SD.

### 2.2 Statistical analysis

SPSS version 22.0 was utilized for conducting the statistical analysis. Quantitative data that followed a normal distribution were presented as $\bar{X}\pm S$ , and a t-test was employed to compare the two groups. For non-normally distributed quantitative data, they were expressed as [*M*(*P*_25_~*P*_75_)], and the Mann-Whitney U test was used for group comparisons. Qualitative data were presented as the number of cases (%) and analyzed using the *χ*^2^ test for group comparisons. The receiver operating characteristic (ROC) curve was employed to determine the optimal cut-off values for CRP and IL-6. Survival analysis was performed using the Kaplan-Meier method for univariate analysis, and the Cox proportional hazards regression model was used for multivariate analysis.

Finally, the selected variables were utilized to create nomograms for the prediction of 1-, 2-, and 3-year OS and PFS rates in patients with uHCC. Subsequently, the model’s discriminatory power and accuracy were assessed. The C-index was employed to evaluate the model’s discrimination ability, while the calibration curve was used to measure the level of agreement between the probabilities generated by the nomogram and the observed actual probabilities.

In terms of statistical analysis, a significance level of P<0.05 was deemed statistically significant.

## 3 Results

### 3.1 Participant characteristics

A retrospective study of 222 uHCC patients receiving immunotherapy (training cohort (n=124) and validation cohort (n=98)) was conducted from November 2020 to May 2023 (**Table 1**). The median age of participants was 54.66 ± 11.27 years, with 85.14% of them were male. Type O blood was found in 40.99% of the patients. The majority of patients (81.08%) had an ECOG score of 1. Most participants had Child-Pugh A grade (62.61%) and were classified as Barcelona Clinical Liver Cancer (BCLC) B stage (63.51%). Hepatitis B was identified as the most common cause of HCC (71.62%), and cirrhosis was present in 46.4% of the patients. Serum AFP< 400 was observed in 154 patients (69.37%). Extrahepatic metastasis was found in 34 patients (15.32%), and microvascular invasion (MVI) was observed in 69 patients (31.08%). The majority of patients (90.09%) received targeted therapy combined with immunotherapy, while a smaller proportion (9.91%) received immune monotherapy. Additionally, 38.29% of patients were treated with thymosin, IL-2, and other immune enhancers. A significant number of participants (59.91%) had previously undergone at least one local treatment for HCC.

**Table 1:** Baseline clinical characteristics of the patients.

Variable	All	training cohort(n=124)	validation cohort(n=98)	*t*/*χ*^2^	*P*
Age±SD(year)	54.66±11.27	53.90±11.28	55.63±11.23	-1.142	0.255
Gender				0.296	0.586
Male	189(85.14)	107(86.29)	82(83.67)		
Female	33(14.86)	17(13.71)	16(16.33)		
Blood type				0.052	0.820
non O-type blood	131(59.01)	74(59.68)	57(58.16)		
O-type blood	91(40.99)	50(40.32)	41(41.84)		
ECOG performance score				3.550	0.060
0	180(81.08)	106(85.48)	74(75.51)		
1	42(18.92)	18(14.52)	24(24.49)		
HBV				0.431	0.512
Absent	63(28.38)	33(26.61)	30(30.61)		
Present	159(71.62)	91(73.39)	68(69.39)		
Cirrhosis				0.158	0.691
Absent	119(53.6)	65(52.42)	54(55.1)		
Present	103(46.4)	59(47.58)	44(44.9)		
Child-Pugh class				1.876	0.391
5	43(19.37)	28(22.58)	15(15.31)		
6	96(43.24)	51(41.13)	45(45.92)		
7	83(37.39)	45(36.29)	38(38.78)		
ALBI grade				–	0.020
1	63(28.38)	28(22.58)	35(35.71)		
2	152(68.47)	94(75.81)	58(59.18)		
3	7(3.15)	2(1.61)	5(5.1)		
AFP level				0.765	0.382
<400 ng/ml	154(69.37)	89(71.77)	65(66.33)		
≥400 ng/ml	68(30.63)	35(28.23)	33(33.67)		
BCLC stage				0.599	0.439
B	141(63.51)	76(61.29)	65(66.33)		
C	81(36.49)	48(38.71)	33(33.67)		
Extrahepatic spread				0.000	0.997
Absent	188(84.68)	105(84.68)	83(84.69)		
Present	34(15.32)	19(15.32)	15(15.31)		
MVI				1.758	0.185
Absent	153(68.92)	90(72.58)	63(64.29)		
Present	69(31.08)	34(27.42)	35(35.71)		
Treatment method				2.819	0.093
targeted therapy+immunotherapy	200(90.09)	108(87.10)	92(93.88)		
immunotherapy	22(9.91)	16(12.90)	6(6.12)		
Prior local therapy for HCC				0.039	0.844
Absent	89(40.09)	49(39.52)	40(40.82)		
Present	133(59.91)	75(60.48)	58(59.18)		
immunopotentiator				0.475	0.491
Absent	137(61.71)	79(63.71)	58(59.18)		
Present	85(38.29)	45(36.29)	40(40.82)		
IL-6				0.658	0.417
<19.82	143(64.41)	77(62.1)	66(67.35)		
≥19.82	79(35.59)	47(37.9)	32(32.65)		
CRP				0.072	0.789
<15.50	136(61.26)	75(60.48)	61(62.24)		
≥15.50	86(38.74)	49(39.52)	37(37.76)		

The validation cohort did not exhibit any notable disparities in basic demographic characteristics, including age, gender, blood type, and tumor biology, when compared to the training cohort. The training cohort demonstrated an ORR of 29.03% and a median follow-up period of 13.5 months. Similarly, the validation cohort displayed an ORR of 27.55% with a median follow-up duration of 13.7 months.

### 3.2 The optimal cut-off values of IL-6 and CRP were determined by the training cohort

We conducted a comparison of baseline serum cytokine levels, laboratory markers, and clinical features between participants in the R and NR groups (**Table 2**). Among the various markers, it was observed that patients in the R group were significantly older than those in the NR group (56.67 vs. 50.42 years, p=0.002). Additionally, there was a higher proportion of males in the R group compared to the NR group (94.2% vs. 76.36%, p=0.004), as well as a greater number of patients with blood type O (49.28% vs. 29.09%, p=0.023). Furthermore, the R group exhibited a higher percentage of patients with AFP< 400 (79.71% vs. 61.82%, p=0.028) and a greater proportion of patients in BCLC B stage (73.91% vs. 45.45%, p=0.001). Conversely, the NR group had a higher incidence of MVI (38.18% vs. 18.84%, p=0.016). Moreover, a larger percentage of patients in the R group had received local treatment (72.46% vs. 45.45%, p=0.002).

**Table 2:** Baseline clinical characteristics of two groups in the training cohort.

Variable	All	R(n=69)	NR(n=55)	*t*/*Z*/*χ*^2^	*P*
Age±SD(year)	53.90±11.28	56.67±9.88	50.42±12.05	3.174	0.002
Gender				8.223	0.004
Male	107(86.29)	65(94.2)	42(76.36)		
Female	17(13.71)	4(5.80)	13(23.64)		
Blood type				5.182	0.023
non O-type blood	74(59.68)	35(50.72)	39(70.91)		
O-type blood	50(40.32)	34(49.28)	16(29.09)		
ECOG performance score				0.000	0.993
0	106(85.48)	59(85.51)	47(85.45)		
1	18(14.52)	10(14.49)	8(14.55)		
HBV				0.311	0.577
Absent	33(26.61)	17(24.64)	16(29.09)		
Present	91(73.39)	52(75.36)	39(70.91)		
Cirrhosis				0.090	0.764
Absent	65(52.42)	37(53.62)	28(50.91)		
Present	59(47.58)	32(46.38)	27(49.09)		
Child-Pugh class				0.609	0.737
5	28(22.58)	16(23.19)	12(21.82)		
6	51(41.13)	30(43.48)	21(38.18)		
7	45(36.29)	23(33.33)	22(40.00)		
ALBI grade				–	1.000
1	28(22.58)	16(23.19)	12(21.82)		
2	94(75.81)	52(75.36)	42(76.36)		
3	2(1.61)	1(1.45)	1(1.82)		
AFP level				4.836	0.028
<400 ng/ml	89(71.77)	55(79.71)	34(61.82)		
≥400 ng/ml	35(28.23)	14(20.29)	21(38.18)		
BCLC stage				10.447	0.001
B	76(61.29)	51(73.91)	25(45.45)		
C	48(38.71)	18(26.09)	30(54.55)		
Extrahepatic spread				3.214	0.073
Absent	105(84.68)	62(89.86)	43(78.18)		
Present	19(15.32)	7(10.14)	12(21.82)		
MVI				5.753	0.016
Absent	90(72.58)	56(81.16)	34(61.82)		
Present	34(27.42)	13(18.84)	21(38.18)		
Treatment method				0.003	0.958
targeted therapy+immunotherapy	108(87.10)	60(86.96)	48(87.27)		
immunotherapy	16(12.90)	9(13.04)	7(12.73)		
Prior local therapy for HCC				9.341	0.002
Absent	49(39.52)	19(27.54)	30(54.55)		
Present	75(60.48)	50(72.46)	25(45.45)		
immunopotentiator				3.476	0.062
Absent	79(63.71)	39(56.52)	40(72.73)		
Present	45(36.29)	30(43.48)	15(27.27)		
IL6,median,median (IQR)	14.96(7.57, 36.55)	9.94(6.93, 16.10)	36.85(20.65, 63.93)	-5.912	<0.001
CRP,median (IQR)	12.40(7.78, 27.65)	9.90(6.40, 13.00)	24.50(10.60, 35.00)	-4.492	<0.001

The levels of serum IL-6 and CRP collected before the first cycle of ICIs injections in the R group were found to be significantly lower compared to the NR group (9.94 vs. 36.85 pg/ml, p<0.001; 9.90 vs. 24.50 mg/L, p <0.001).

**Figure 2:**
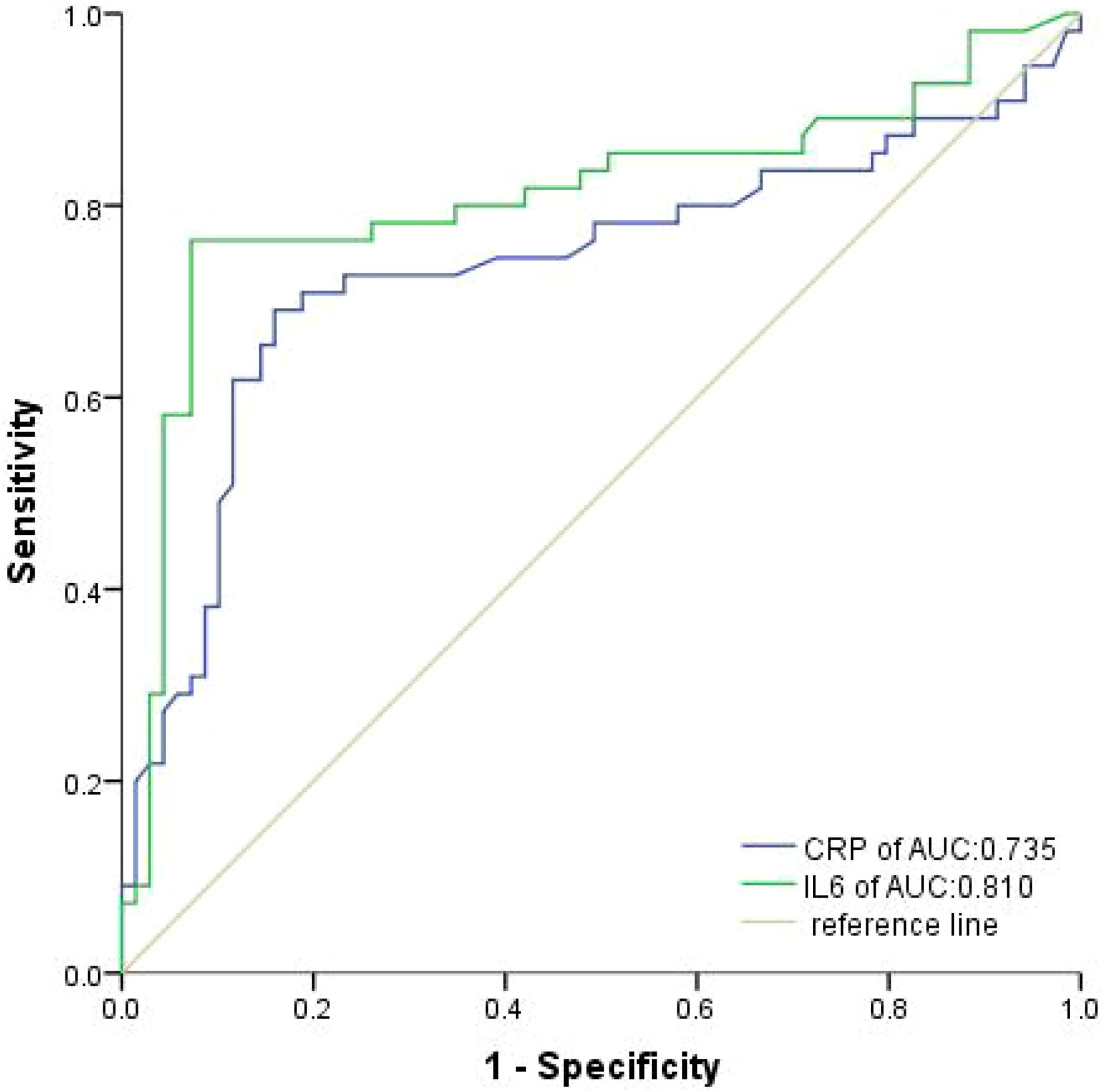
Receiver operating characteristic curves were produced to assess the discriminative power of IL-6 and CRP in the training cohort.

According to the ROC analysis, the optimal cutoff values for IL-6 and CRP were determined to be 19.82pg/ml and 15.50 mg/L, respectively, in both groups (**Figure 2**). At baseline, a majority of participants, specifically 62.10% and 60.48%, were found to have low levels of IL-6 and CRP, respectively.

Using these determined cutoff values, 35.59% of participants in this study were classified as having high IL-6, with 21.17% in the training cohort and 14.41% in the validation cohort (**Table 3**). Additionally, 38.74% of participants in this study were identified as having high CRP, with 22.07% in the training cohort and 16.67% in the validation cohort (**Table 4**).

**Table 3:** Clinical characteristics based on IL-6 status.

Variable	All	IL-6-low(n=143)	IL-6-high(n=79)	t/*χ*^2^	*P*
Age±SD(year)	54.66±11.27	55.52±10.69	53.10±12.16	1.539	0.125
Gender				0.010	0.919
Male	189(85.14)	122(85.31)	67(84.81)		
Female	33(14.86)	21(14.69)	12(15.19)		
Blood type				0461	0.497
non O-type blood	131(59.01)	82(57.34)	49(62.03)		
O-type blood	91(40.99)	61(42.66)	30(37.97)		
ECOG performance score				0.000	0.985
0	180(81.08)	116(81.12)	64(81.01)		
1	42(18.92)	27(18.88)	15(18.99)		
HBV				0.242	0.623
Absent	63(28.38)	39(27.27)	24(30.38)		
Present	159(71.62)	104(72.73)	55(69.62)		
Cirrhosis				0.216	0.642
Absent	119(53.6)	75(52.45)	44(55.7)		
Present	103(46.4)	68(47.55)	35(44.3)		
Child-Pugh class				1.724	0.422
5	43(19.37)	24(16.78)	19(24.05)		
6	96(43.24)	64(44.76)	32(40.51)		
7	83(37.39)	55(38.46)	28(35.44)		
ALBI grade				–	0.002^*^
1	63(28.38)	50(34.97)	13(16.46)		
2	152(68.47)	91(63.64)	61(77.22)		
3	7(3.15)	2(1.40)	5(6.33)		
AFP level				1.337	0.248
<400 ng/ml	154(69.37)	103(72.03)	51(64.56)		
≥400 ng/ml	68(30.63)	40(27.97)	28(35.44)		
BCLC stage				5.668	0.017
B	141(63.51)	99(69.23)	42(53.16)		
C	81(36.49)	44(30.77)	37(46.84)		
Extrahepatic spread				0.668	0.414
Absent	188(84.68)	119(83.22)	69(87.34)		
Present	34(15.32)	24(16.78)	10(12.66)		
MVI				12.019	0.001
Absent	153(68.92)	110(76.92)	43(54.43)		
Present	69(31.08)	33(23.08)	36(45.57)		
Treatment method				2.214	0.137
targeted therapy+immunotherapy	200(90.09)	132(92.31)	68(86.08)		
immunotherapy	22(9.91)	11(7.69)	11(13.92)		
Prior local therapy for HCC				8.729	0.003
Absent	89(40.09)	47(32.87)	42(53.16)		
Present	133(59.91)	96(67.13)	37(46.84)		
immunopotentiator				0.420	0.517
Absent	137(61.71)	86(60.14)	51(64.56)		
Present	85(38.29)	57(39.86)	28(35.44)		
CRP				66.771	<0.001
<15.50	136(61.26)	116(81.12)	20(25.32)		
≥15.50	86(38.74)	27(18.88)	59(74.68)		

* indicates that the test method is Fisher exact probability method.

**Table 4:** Clinical characteristics based on CRP status.

Variable	All	CRP-low(n=136)	CRP-high(n=86)	*t*/*χ*^2^	*P*
Age±SD(year)	54.66±11.27	55.09±10.90	53.99±11.87	0.708	0.480
Gender				0.477	0.490
Male	189(85.14)	114(83.82)	75(87.21)		
Female	33(14.86)	22(16.18)	11(12.79)		
Blood type				3.067	0.080
non O-type blood	131(59.01)	74(54.41)	57(66.28)		
O-type blood	91(40.99)	62(45.59)	29(33.72)		
ECOG performance score				0.370	0.543
0	180(81.08)	112(82.35)	68(79.07)		
1	42(18.92)	24(17.65)	18(20.93)		
HBV				0.184	0.668
Absent	63(28.38)	40(29.41)	23(26.74)		
Present	159(71.62)	96(70.59)	63(73.26)		
Cirrhosis				0.092	0.761
Absent	119(53.6)	74(54.41)	45(52.33)		
Present	103(46.4)	62(45.59)	41(47.67)		
Child-Pugh class				0.108	0.948
5	43(19.37)	26(19.12)	17(19.77)		
6	96(43.24)	58(42.65)	38(44.19)		
7	83(37.39)	52(38.24)	31(36.05)		
ALBI grade				–	0.151^*^
1	63(28.38)	42(30.88)	21(24.42)		
2	152(68.47)	92(67.65)	60(69.77)		
3	7(3.15)	2(1.47)	5(5.81)		
AFP level				5.238	0.022
<400 ng/ml	154(69.37)	102(75)	52(60.47)		
≥400 ng/ml	68(30.63)	34(25)	34(39.53)		
BCLC stage				4.758	0.029
B	141(63.51)	94(69.12)	47(54.65)		
C	81(36.49)	42(30.88)	39(45.35)		
Extrahepatic spread				0.489	0.484
Absent	188(84.68)	117(86.03)	71(82.56)		
Present	34(15.32)	19(13.97)	15(17.44)		
MVI				11.255	0.001
Absent	153(68.92)	105(77.21)	48(55.81)		
Present	69(31.08)	31(22.79)	38(44.19)		
Treatment method				0.464	0.496
targeted therapy+immunotherapy	200(90.09)	124(91.18)	76(88.37)		
immunotherapy	22(9.91)	12(8.82)	10(11.63)		
Prior local therapy for HCC				4.472	0.034
Absent	89(40.09)	47(34.56)	42(48.84)		
Present	133(59.91)	89(65.44)	44(51.16)		
immunopotentiator				0.000	0.984
Absent	137(61.71)	84(61.76)	53(61.63)		
Present	85(38.29)	52(38.24)	33(38.37)		
IL6				66.771	<0.001
<19.82	143(64.41)	116(85.29)	27(31.40)		
≥19.82	79(35.59)	20(14.71)	59(68.60)		

* indicates that the test method is Fisher exact probability method.

### 3.3 The correlation between IL6, CRP, and clinical characteristics

In our study, we have successfully demonstrated that an increase in IL-6 levels is significantly associated with a high ALBI score (p=0.002), BCLC stage (46.84% vs. 30.77%, p=0.017), and the presence of MVI (45.57% vs. 23.08%, p=0.001) (**Table 3**).

Furthermore, elevated levels of CRP were found to be associated with high AFP levels (39.53% vs. 25%, p=0.022), advanced BCLC stages (45.35% vs. 30.88%, p=0.029), and the presence of MVI (44.19% vs. 22.79%, p=0.001) (**Table 4**).

### 3.4 Survival analysis

**Figure 3:**
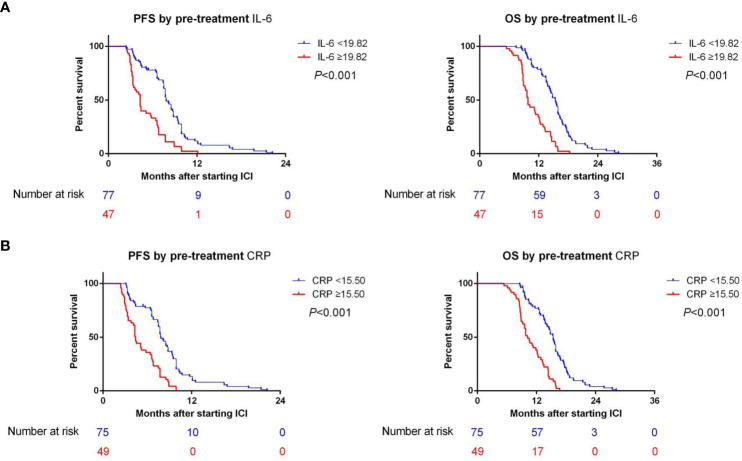
**(A)** The Kaplan-Meier survival curves demonstrated a significant correlation between IL-6 levels and both PFS and OS in training cohort. **(B)** The Kaplan-Meier survival curves demonstrated a significant correlation between CRP levels and both PFS and OS in training cohort.

**Figure 4:**
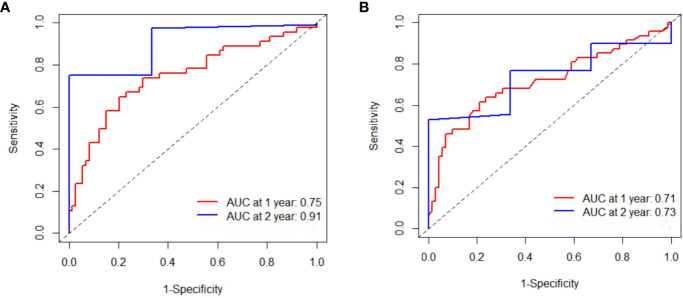
Time-dependent Reciever operating characteristics curves were produced for IL-6 **(A)** and CRP **(B)** at 1-, 2-year, respectively in training cohort.

In the training cohort, the median PFS (mPFS) was found to be significantly longer in the low-IL-6 group compared to the high-IL-6 group (7.8 vs. 4.3 mo, HR 2.884, 95%CI 1.939-4.289, p<0.001), as indicated by the Kaplan-Meier survival curve. Additionally, the median OS (mOS) was significantly prolonged in the low-IL-6 group compared to the high-IL-6 group (15.4 vs. 9.8 mo, HR 3.518, 95%CI 2.338-5.295, p<0.001) (**Figure 3A**). The AUC values at 1-, and 2-year of follow-up were 0.75, and 0.91, respectively (**Figure 4A**), indicating good sensitivity and specificity of IL-6.

Furthermore, when comparing patients in the low CRP group to those in the high CRP group, it was observed that the low CRP group had significantly longer mPFS (7.8 vs. 4.4 mo, HR 2.845, 95%CI 1.913-4.232, p<0.001) and significantly longer mOS (15.4 vs. 10.2 mo, HR 3.586, 95%CI 2.357-5.455, p<0.001) (**Figure 3B**).The AUC values at 1-, and 2-year of follow-up were 0.71, and 0.73, respectively (**Figure 4B**).

In order to account for potential confounding factors in survival outcomes, such as Child-Pugh scores, we conducted a multivariate Cox proportional risk model to further validate the clinical impact of baseline IL-6 and CRP levels. The results of the multivariate analysis revealed that high baseline IL-6 levels (HR 1.624, 95%CI 1.016-2.596, p=0.043), high CRP levels (HR 1.709, 95%CI 1.041-2.807, p=0.034), and MVI (HR 1.751, 95%CI 1.059-2.894, p=0.029) remained significant factors associated with poor PFS (**Table 5**).

**Table 5:** Cox proportional hazards analysis of factors related to PFS in training cohort.

Variable	(n=124)	Univariate analysis		Multivariate analysis	
		HR(95%CI)	*P*	HR(95%CI)	*P*
Blood type(non-O/O)	74/50	0.650(0.446~0.948)	0.025	0.716(0.487~1.054)	0.090
ECOG performance score(0/1)	106/18	1.461(0.880~2.426)	0.142		
HBV (Absent/Present)	33/91	0.835(0.558~1.250)	0.381		
Cirrhosis (Absent/Present)	65/59	1.070(0.745~1.538)	0.713		
Child-Pugh class(5-6/7)	79/45	1.369(0.943~1.987)	0.099		
ALBI grade(1/2-3)	28/96	0.848(0.553~1.302)	0.452		
AFP(<400/≥400)	89/35	1.539(1.031~2.297)	0.035	1.239(0.820~1.873)	0.308
BCLC stage(B/C)	76/48	0.767(0.530~1.108)	0.158		
Extrahepatic spread(Absent/Present)	105/19	1.093(0.668~1.789)	0.724		
MVI(Absent/Present)	90/34	2.781(1.806~4.281)	<0.001	1.751(1.059~2.894)	0.029
IL6(<19.82/≥19.82)	77/47	2.884(1.939~4.289)	<0.001	1.624(1.016~2.596)	0.043
CRP(<15.50/≥15.50)	75/49	2.845(1.913~4.232)	<0.001	1.709(1.041~2.807)	0.034

Furthermore, high baseline IL-6 levels (HR 2.146, 95%CI 1.361-3.383, p=0.001), high CRP levels (HR 1.846, 95%CI 1.128-3.022, p=0.015), non-O blood type (HR 0.523, 95%CI 0.346-0.789, p=0.002), cirrhosis (HR 1.499, 95%CI 1.012-2.220, p=0.043), and high AFP (HR 2.490, 95%CI 1.607-3.859, p<0.001) were still found to be significant factors associated with poor OS (**Table 6**). At the conclusion of the follow-up period in August 2023, a total of 38.4% of patients had succumbed to their condition. The mPFS was determined to be 7.3 months (95%CI: 6.846-7.754), while the mOS was found to be 13.6 months (95%CI: 12.691-14.509).

**Table 6:** Cox proportional hazards analysis of factors related to OS in training cohort.

Variable	(n=124)	Univariate analysis		Multivariate analysis	
		HR(95%CI)	*P*	HR(95%CI)	*P*
Blood type(non-O/O)	74/50	0.463(0.314~0.685)	<0.001	0.523(0.346~0.789)	0.002
ECOG performance score(0/1)	106/18	0.995(0.600~1.649)	0.983		
HBV (Absent/Present)	33/91	0.851(0.568~1.274)	0.433		
Cirrhosis (Absent/Present)	65/59	1.538(1.056~2.239)	0.025	1.499(1.012~2.220)	0.043
Child-Pugh class(5-6/7)	79/45	1.419(0.973~2.071)	0.069		
ALBI grade(1/2-3)	28/96	0.836(0.538~1.299)	0.426		
AFP(<400/≥400)	89/35	2.295(1.509~3.491)	<0.001	2.490(1.607~3.859)	<0.001
BCLC stage(B/C)	76/48	1.052(0.723~1.530)	0.792		
Extrahepatic spread(Absent/Present)	105/19	1.385(0.841~2.283)	0.201		
MVI(Absent/Present)	90/34	2.198(1.440~3.355)	<0.001	1.530(0.955~2.451)	0.077
IL6(<19.82/≥19.82)	77/47	3.518(2.338~5.295)	<0.001	2.146(1.361~3.383)	0.001
CRP(<15.50/≥15.50)	75/49	3.586(2.357~5.455)	<0.001	1.846(1.128~3.022)	0.015

### 3.5 Creation and validation of nomograms

**Figure 5:**
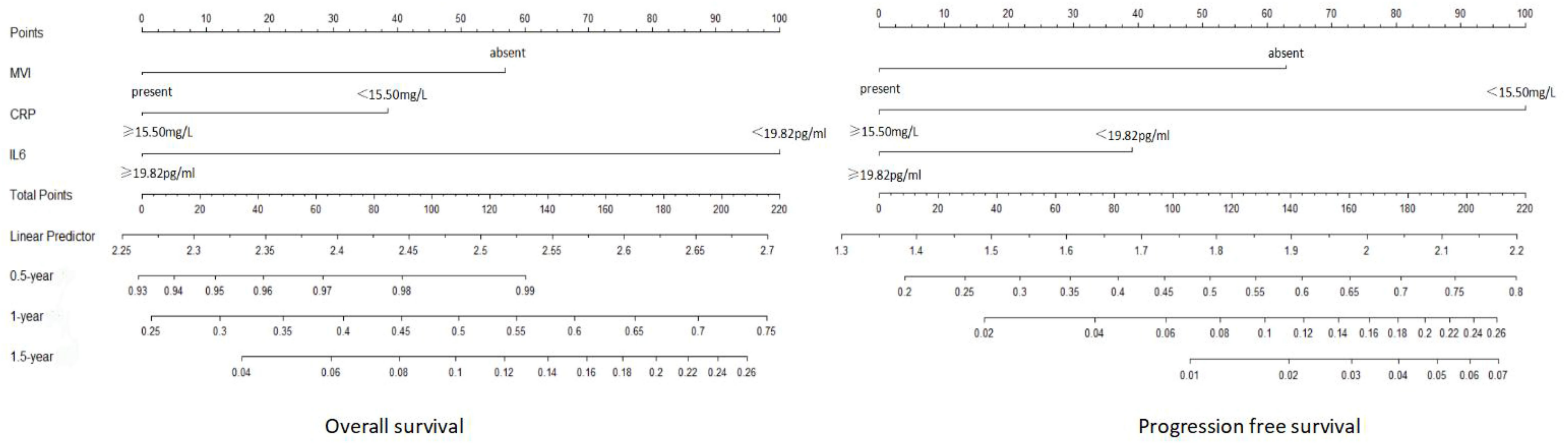
Establishment of OS and PFS nomograms in validation cohort.

Hence, we utilized MVI, IL-6, and CRP as the three variables in the advanced HCC nomogram to predict patient survival (**Figure 5**). By calculating the sum of scores for these variables using a column chart, we can estimate the 1-year, 2-year, and 3-year OS rates, as well as the PFS rate for advanced HCC patients. To assess the model’s performance, we employed the C-index and calibration curve. In the training group, the C-index for the OS-based prediction model was 0.796 (95%CI 0.75386-0.83814) and 0.741 (95%CI 0.68024-0.80176) in the validation group. For the PFS-based prediction model, the C-index was 0.796 (95%CI 0.74994-0.84206) and 0.761 (95%CI 0.70808-0.81392) in the respective groups. These findings indicate that the prognostic model effectively identifies the survival rate of patients with advanced HCC.

**Figure 6:**
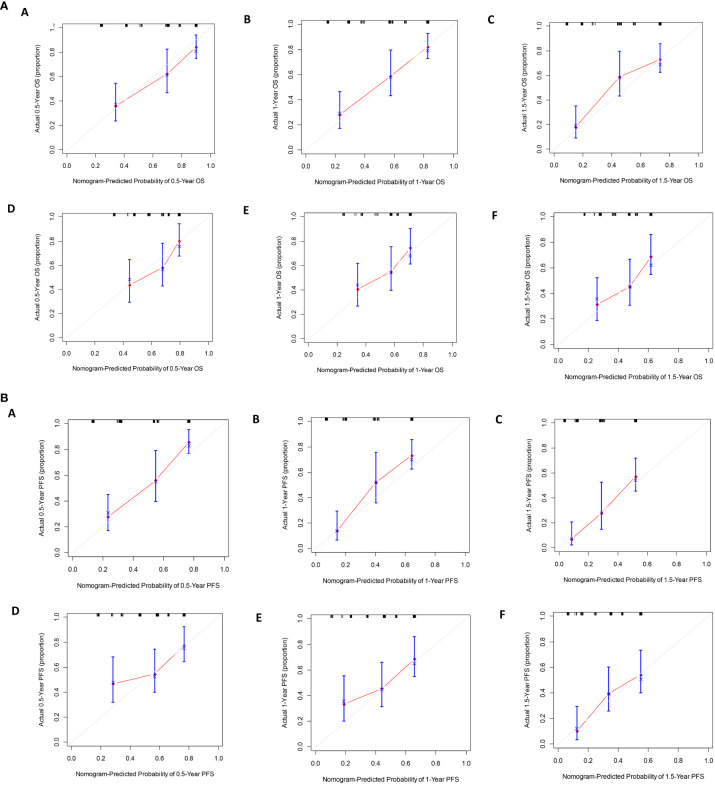
**(A)** The calibration curves for predicting OS at **(A)** 0.5-year and **(B)** 1-year and **(C)** 1.5-year in the training cohort, and at **(D)** 0.5-year **(E)** 1-year and **(F)** 1.5-year in the validation cohort. **(B)**. The calibration curves for predicting PFS at **(A)** 0.5-year and **(B)** 1-year and **(C)** 1.5-year in the training cohort, and at **(D)** 0.5-year **(E)** 1-year and **(F)** 1.5-year in the validation cohort.

**Figure 7:**
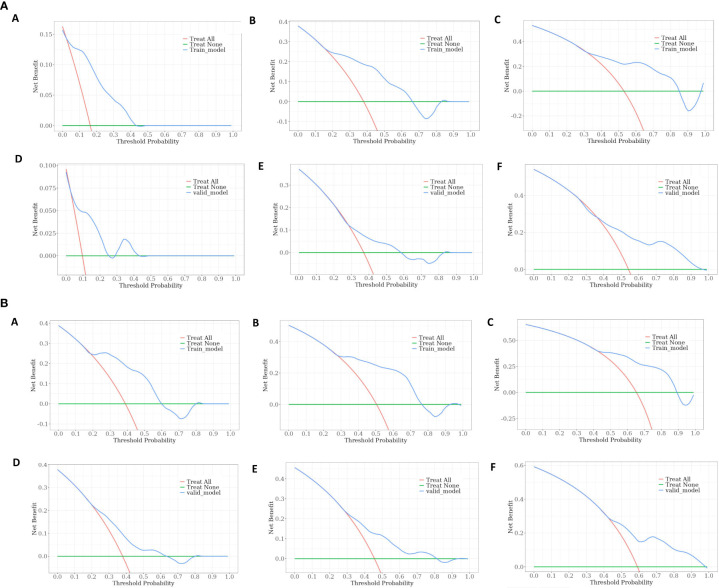
**(A)** Decision curves of the nomogram predicting OS at **(A)** 0.5-year and **(B)** 1-year and **(C)** 1.5-year in the training cohort, and at **(D)** 0.5-year **(E)** 1-year and **(F)** 1.5-year in the validation cohort. **(B)**. Decision curves of the nomogram predicting PFS at **(A)** 0.5-year and **(B)** 1-year and **(C)** 1.5-year in the training cohort, and at **(D)** 0.5-year **(E)** 1-year and **(F)** 1.5-year in the validation cohort.

The calibration curve prediction models demonstrate a high level of concordance between the predicted probabilities and the actual 0.5-year, 1-year, and 1.5-year OS rates and PFS rates for both the training and validation groups (**Figure 6**). Decision curves of the nomogram are showed to predict 0.5-year, 1-year, and 1.5-year OS rates and PFS rates for both the training and validation groups (**Figure 7**). This finding underscores the robust clinical predictive capability of the nomograms.

### 3.6 Reaction effect

**Figure 8:**
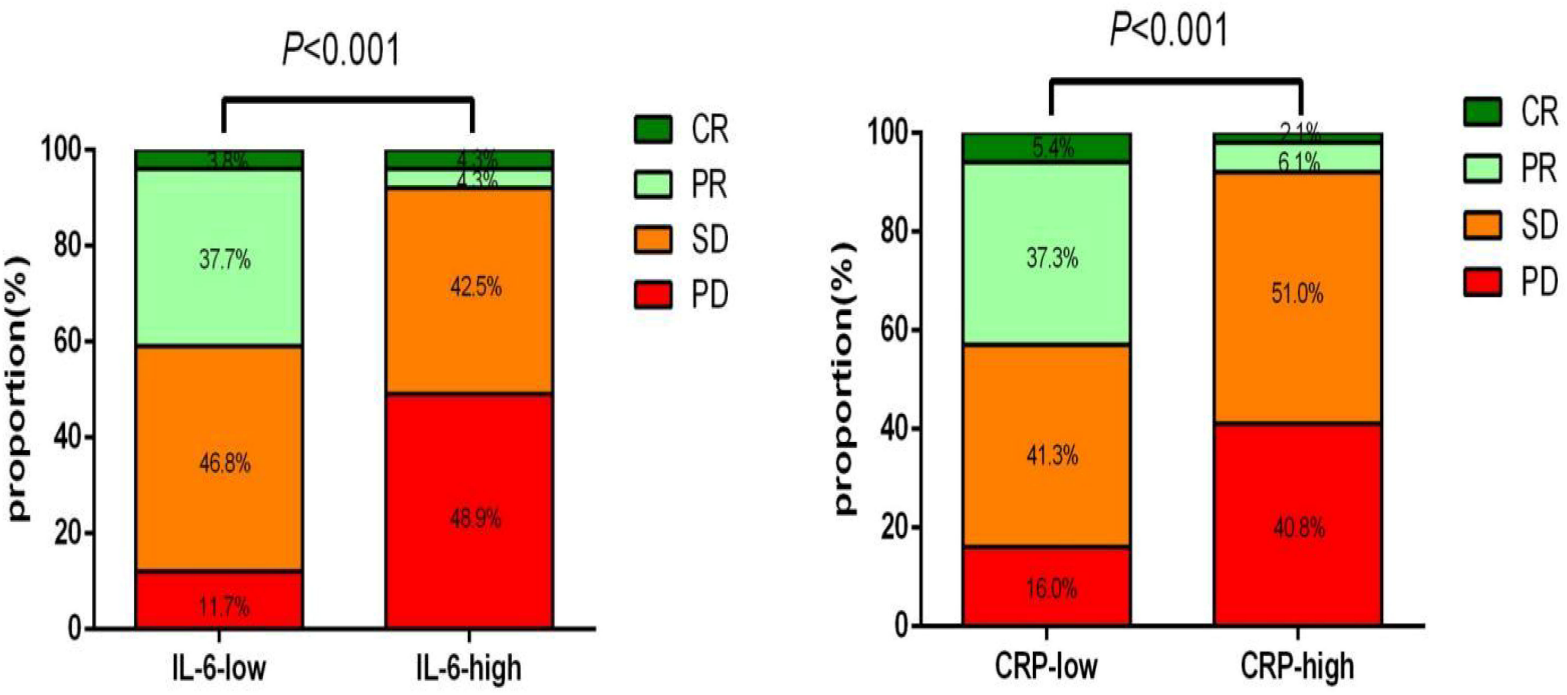
The response effect to the treatment according to IL-6 and CRP in training cohort.

In the study, a total of 222 patients were included, and the ORR was found to be 28.38% (63/222). In the training cohort, a significantly greater proportion of patients in the low IL6 group achieved ORR at baseline compared to those in the high IL6 group (41.56% vs. 8.51%, p<0.001). Similarly, patients in the low CRP group had a significantly higher ORR compared to those in the high CRP group (42.67% vs. 8.16%, p<0.001) (**Figure 8**).

### 3.7 The levels of serum IL-6 and CRP exhibited a correlation

**Figure 9:**
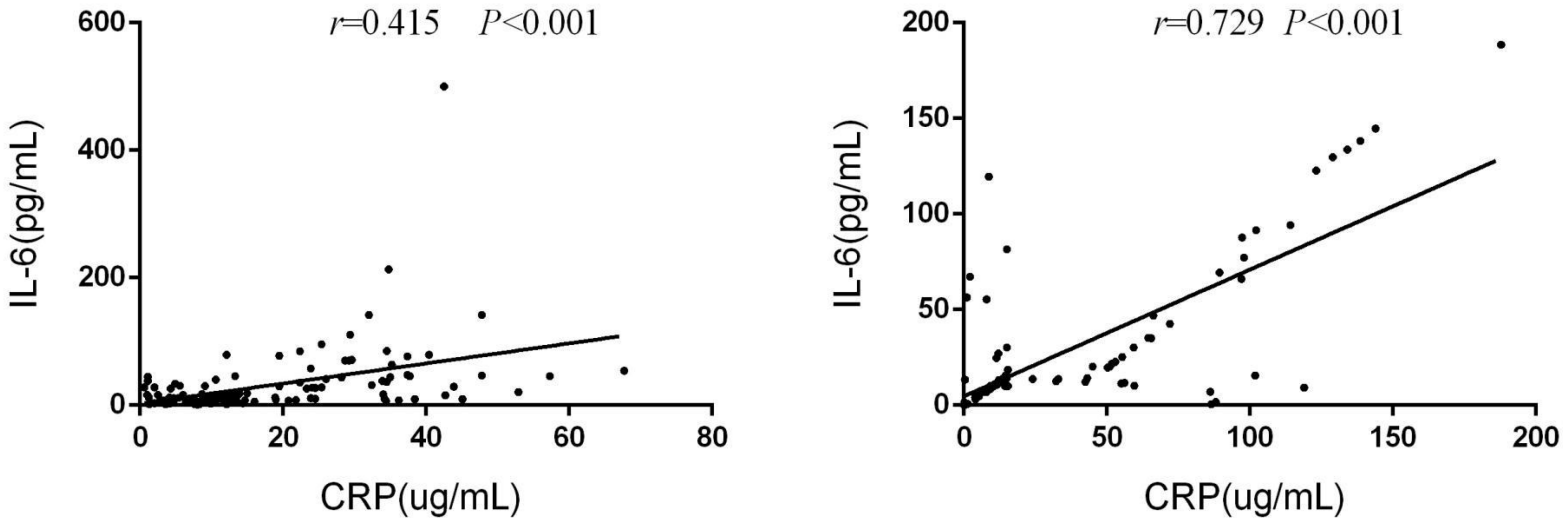
IL-6 and CRP serum levels are correlated in training cohort and validation cohort.

In the training group, there was a significant correlation between serum IL-6 and CRP levels at baseline (r=0.415, p<0.001). This correlation was similarly observed in the validation group (r=0.729, p<0.001)(**Figure 9**).

## 4 Discussion

Immunotherapy has emerged as the primary treatment option for uHCC. However, a subset of patients still exhibit resistance to immunotherapy, posing a challenge for clinicians in determining the most appropriate course of action. In this study, we have identified a small proportion (26.58%) of unresectable HCC patients who possess elevated baseline levels of IL-6 and CRP, which are associated with diminished clinical benefits from immunotherapy. Notably, these findings remained significant even after accounting for various confounding factors such as age, sex, ECOG performance status, Child-Pugh classification, AFP levels, presence of MVI, and extrahepatic metastases.

PD-L1 expression, microsatellite instability (MSI), and tumor mutation load (TMB) have been established as reliable predictive markers for immunotherapy response in various solid tumors. However, their clinical applicability in HCC remains uncertain (14). Furthermore, since obtaining tumor tissue through biopsy is not obligatory for HCC diagnosis, it cannot be routinely utilized as a predictive biomarker. Consequently, the potential of non-invasive circulating biomarkers to accurately forecast the effectiveness of immunotherapy in HCC is highly promising.

In this study, a substantial body of evidence was employed to establish the clinical significance of IL-6 and CRP. Initially, it was observed that patients who exhibited elevated levels of IL-6 and CRP at baseline experienced unfavorable survival outcomes subsequent to immunotherapy treatment. These findings were subsequently corroborated through confirmatory cohort analysis and multifactor analysis. Furthermore, it was determined that patients with elevated levels of IL-6 and baseline CRP displayed reduced responsiveness to immunotherapy and encountered challenges in achieving the anticipated outcomes of conversion surgery. Lastly, a strong correlation between IL-6 and CRP levels was identified, suggesting that these biomarkers may contribute to the poor prognosis observed in patients.

Local treatments such as RFA and TACE are believed to have the ability to eliminate the primary tumor, release tumor antigens, activate both innate and adaptive immunity, and enhance anti-tumor immunity that is hindered by immune checkpoints (15). In turn, the use of immune checkpoint inhibitors can further augment this effect, resulting in a synergistic immunogenic cell death (16). Interestingly, it has been observed that higher levels of immunity are associated with improved efficacy, which may be contrary to our view. However, there are plausible explanations for this phenomenon. Firstly, not all local treatments are beneficial for anti-tumor immunity, as they can induce hypoxia, increased vascular permeability, and the release of certain cytokines (such as VEGF and TGFβ) that inhibit the effectiveness of anti-tumor immune responses (17). Secondly, the inflammatory response triggered by local treatment follows a cyclical pattern, with elevated levels of immune factors in the initial week after treatment and subsequent decline below baseline levels over time. Based on this observation, it is hypothesized that it would be optimal to delay the initiation of local treatment by at least one week before commencing immune checkpoint inhibitors.

Furthermore, our research suggests that individuals with blood type O exhibit a favorable prognosis when undergoing ICIs treatment for HCC. Prior investigations have established the ABO antigen as a cancer-associated antigen. Okada et al. discovered structural similarities between certain antigens in HCC tissues and ABO antigens (18). Consequently, patients with blood types other than O may experience a compromised tumor immune response, as their immune systems may possess diminished capacity to identify and combat tumor cells expressing antigens that bear resemblance to ABO antigens (19).

The established IL-6 cutoff values of 19.82 pg/ml and CRP cutoff values of 15.5 mg/L were utilized to assess the effectiveness of immunotherapy for uHCC. A separate study conducted recently discovered a lower IL-6 cutoff value of 3.2 pg/ml for predicting the efficacy of combination therapy involving Atezolizumab and Bevacizumab in HCC (20). This finding implies that further investigation is warranted through larger-scale studies to validate the results.

IL-6, a proinflammatory cytokine, plays a crucial role in the pathogenesis of various acute and chronic inflammatory conditions (21), including liver diseases such as cirrhosis and liver cancer (22). Experimental studies using a diethylnitrosamine-induced hepatocellular carcinoma model have demonstrated that IL-6 promotes compensatory proliferation of hepatocytes, and its absence or inhibition of IL-6 receptor-related proteins impedes liver tumor development (23). Clinical evidence from a meta-analysis of 18 studies involving patients with HCC and hepatitis revealed a progressive elevation in serum IL-6 levels with disease progression, from healthy individuals to hepatitis, cirrhosis, and ultimately HCC (7). CRP, a systemic marker of inflammation, has been associated with prognosis and clinical outcomes in various malignancies and in patients undergoing immunotherapy. A scoring system called CRAFITY, based on CRP and AFP levels, was developed using data from 190 patients with advanced HCC who received single or combined immunotherapy. The results indicated that baseline AFP levels ≥100ng/ml and CRP levels≥1 mg/dl were independently associated with OS in these patients (24). However, it is important to consider that CRP levels can be influenced by injury or infection. A study focusing on non-small cell lung cancer patients found that dynamic changes in CRP levels, specifically an initial increase followed by a decrease, were predictive of a positive response to immunotherapy and improved OS. Notably, this predictive ability was observed with only four weeks of CRP monitoring (25). In our study, we also observed that HCC patients with elevated plasma IL-6 and CRP levels tended to have more advanced disease according to the BCLC staging system and a higher incidence of MVI, suggesting a positive correlation between circulating IL-6 and CRP levels and HCC progression. Furthermore, HCC patients with high CRP levels also exhibited elevated AFP levels, which can serve as an indicator of the aggressive nature of HCC.

Recent studies have suggested a potential role for IL-6 in mediating drug resistance in immunotherapy (8, 26). Specifically, IL-6 has been found to activate STAT3 in dendritic cells, resulting in the downregulation of major histocompatibility complex Class II expression (27). Additionally, IL-6 has been shown to recruit bone marrow-derived suppressor cells (MDSCs), which suppress the immune response to tumor antigens and inhibit T cells, including those involved in HCC (28). MDSCs have been implicated in blocking the anticancer activity of ICIs (29). Preclinical research has demonstrated that blocking IL-6 can inhibit tumor growth by enhancing the activity of CD4+/CD8+ effector T cells while suppressing Th17 and macrophages (8). Building upon these findings, an ongoing Phase Ib/II clinical trial (NCT04524871) is currently evaluating the efficacy and safety of adding anti-IL-6 therapy to the treatment of unresectable HCC patients receiving Ate/Bev. Furthermore, CRP stimulation of T cells has been shown to influence cytokine secretion, promoting IL-4 secretion and inhibiting IFN-γ secretion, thereby directly impacting Th1/Th2 differentiation. Consequently, it would be worthwhile to further investigate potential mechanistic interactions between IL-6, CRP signaling, and the efficacy of ICIs.

The study has several limitations that should be acknowledged. Firstly, it is important to note that this study was conducted at a single center and is retrospective in nature. Therefore, it is necessary to validate the findings externally in other centers to ensure their generalizability. Additionally, it is worth considering that the levels of IL-6 and CRP in HCC may be influenced by various factors, such as the deterioration of liver function, the progression of HCC staging, and the immunophenotype of HCC. Consequently, further analyses stratified by these factors are required to better understand the underlying causes of elevated IL-6 levels. Furthermore, it is important to acknowledge that the study cohort in this research was relatively small, the observation period was not sufficiently long, and there was a lack of longitudinal comparative analysis before and after immunotherapy.

## 5 Summary

In conclusion, the elevated baseline serum levels of IL-6 and CRP has been found to be associated with unfavorable clinical outcomes in patients with uHCC who undergo immunotherapy. It is recommended that clinicians conduct early response assessments in patients with high baseline IL-6 and CRP levels during treatment. However, it is important not to exclude these patients from receiving potentially effective standard care. Instead, further comprehensive investigations should be conducted to enhance the efficacy of immunotherapy in this patient population. Moreover, the detection of IL-6 and CRP should be standardized to optimize the management of predicting immunotherapy efficacy. Additionally, future research should focus on elucidating the underlying mechanisms linking high IL-6 and CRP levels to reduced clinical benefits from immunotherapy, with the aim of establishing standardized cutoff values.

## Data availability statement

The original contributions presented in the study are included in the article/supplementary materials, further inquiries can be directed to the corresponding author/s.

## Ethics statement

The studies involving humans were approved by the Tongji hospital’s Ethics Committee (TJ-IRB20230866). The studies were conducted in accordance with the local legislation and institutional requirements. The ethics committee/institutional review board waived the requirement of written informed consent for participation from the participants or the participants’ legal guardians/next of kin because Given the retrospective design of the study, the committee waived the need for informed consent from all patients.

## Author contributions

JD: Conceptualization, Data curation, Writing – original draft. ZH: Conceptualization, Data curation, Writing – original draft. EZ: Conceptualization, Data curation, Writing – original draft.
